# A comparative study of functional outcome of treatment of intra articular fractures of distal radius fixed with percutaneous Kirschner’s wires vs T-plate

**DOI:** 10.12669/pjms.333.11421

**Published:** 2017

**Authors:** Jahangir Iqbal Khan, Faisal Nazeer Hussain, Tahir Mehmood, Omer Adil

**Affiliations:** 1 Jahangir Iqbal Khan, MS Orthopedic, Post Graduate Medical Institute, Lahore General Hospital (LGH), Lahore, Pakistan; 2 Faisal Nazeer Hussain, FCPS Orthopedic, Professor of Orthopedics, Avicenna Medical College, DHA 9, Lahore Cantt, Pakistan; 3 Tahir Mehmood, MS Orthopedic, Assistant Professor, Post Graduate Medical Institute, Lahore General Hospital (LGH), Lahore, Pakistan; 4 Omer Adil, FRCS. Associate Professor, Post Graduate Medical Institute, Lahore General Hospital (LGH), Lahore, Pakistan

**Keywords:** Distal Radius, Intra-articular Fractures, Kirschner’s wires, T-plate

## Abstract

**Background & Objective:**

Fractures of the distal radius are common with a variable prognosis in case of intra articular extension. The available options include Plaster, External fixation, Prefabricated Splintage using Ligamentotaxis, K-wire fixation, and open reduction internal fixation with T-plate without an as yet clear advantage of one over the others. If these fractures are allowed to collapse, radial shortening, angulation and articular incongruity may cause permanent deformity and loss of function. This limited small scale study was intended to compare the functional results of treatment of these fractures with a T plate and K-wires.

**Methods:**

This was a prospective experimental study conducted at department of Orthopedics of PGMI/Lahore General Hospital, Lahore. Total 30 patients were included and randomized into two groups of 15 patients each. Group-A patients were treated with Krischner’s wires and Group-B patients were treated with a T-Plate with open reduction. Informed consent was taken. Post operative follow up was done for 12 weeks for the outcome parameters (Green and O’Brien score).

**Results:**

Mean age of patients in Group-A and B was 36.13±9.81 and 44.73±7.86 years respectively. In Group-A there were 10 male and 5 female patients and in Group-B there were 8 male and seven female patients respectively. In Group-A nine patients presented with Fernandez type-II and six patients presented with Fernandez type-III fracture. While in Group-B 10 patients presented with Fernandez type-II and five patients presented with Fernandez type-III fracture. Among Group-A patient’s final outcome was excellent in 86.67% patients while in Group-B only 53.33% patients had excellent outcome at three months follow up.

**Conclusion:**

Percutaneous Kirschner’s wires appeared to be more effective as compared to T-Plate fixation in terms of functional outcome for treating intra-articular distal radius fractures.

## INTRODUCTION

Distal radius fracture account for a lot of cases in accident and emergency department.^1^,^2^ The common mechanisms of injuries are road traffic accidents, fall from height, industrial and sports trauma.^2^ For a long time plaster casts remained the mainstay of treating intra-articular fractures of distal radius. Due to collapse of the fracture fragments occurs, radial shortening, angulation and articular incongruity that may result in permanent deformity. This loss of reduction causes an unacceptable deformity and relative ulnar lengthening leading to pain over the medial side of the wrist. The earlier nomenclature Colle, Smith, Barton lead to a broader term Distal Radial Fractures DRF covers all intra and extra-articular varieties. Almost all the classification systems face criticism but Fernandez Classification is popular.^3^ The distal radius fractures are classified by Fernandez Classification,^4^,^5^ which divides these fractures into five Types. Despite of its being a common injury there is little agreement on choosing one way of treating all these injuries. Radwan M, et al. 2009 in their study comparing distal radius fractures fixation with K-wires with T-plating, found equivocal results.^6^ Triangular fibro cartilage complex (TFCC) injury is a frequent cause of symptoms in the injured wrist post operatively. Many authors like Koval, 2006^7^, Chen and Jupiter, 2007^8^, Lichtman et al. 2010^9^ have suggested the factors leading to redisplacement of the fracture during treatment as:

- Initial fracture displacement
- Among older patients porotic bones gross comminution can cause a redisplacement
- Metaphyseal comminution.

Redisplacement after a closed manipulation indicates instability at the fracture site and re-manipulation rarely produces a better radiographic outcome^10^ and some suggest a dorsal comminution at the metaphysic is the cause.^11^

Plate fixation holds its merit due to its stability, period of immobilization is short, and early return to previous active life. A locking plate fixation has gained popularity in recent years in management of distal radius fractures. Anatomical restoration of articular surface and fragments alignment promote functional return and avoids early osteoarthritic changes.^2^,^6^,^12^-^14^ There are drawbacks of open reduction like skin scaring, possible injury to tendons, need for a second procedure to remove the plate, a higher cost and requirement of higher technical skills than use of K-wires for percutaneous fixation. In a wide variety of situations volar plates are used to buttress volar fragments and even dorsal fragments are stabilized through this route. K- wires are easy to insert, do minimal tissue damage, have atraumatic insertion, less swelling and stiffness making them a routine preference.^15^-^17^ Other advantages include less chance of infection, fracture healing is better,^15^-^17^ Their drawbacks are lesser rigid fixation, peripheral neurovascular damage and migration of wires. We have conducted this study in randomized, comparative interventional setting prospectively. The purpose was to document scientifically any differences in functional recovery.

## METHODS

Thirty patients were included in the study on-probability convenient sampling with random allocation to study Groups A&B as they presented to emergency and OPD. Only patients with distal radial fractures within one week of injury were taken and patients with injuries elsewhere to the same bone fresh or old were avoided. Patients with open fractures were excluded. The sample size was calculated with 95% confidence interval, 10% margin of error, and magnitude of excellent outcome i.e. 63.2% with K-wire use and 66.6% with T-plate. Both groups were counseled as per IRB requirements and an informed consent was taken. All patients were operated by consultant staff using percutaneously K-wires under c-arm guidance and plaster cast in Group A. Group B was operated through volar approach, under tourniquet control and the use of a volar AO plate. Similar plaster splints, antibiotic (cephalosporins) and analgesic regimens were used in both groups. Stitches were removed in Group B after 10 days and a gentle physiotherapy plan was instituted. Casts were removed at 3-4 weeks in the other group and wires were extracted after 4-6 weeks. A similar rehabilitation program consisting of assisted and active range of motion exercises was done in both the groups for three months.

Functional recovery was measured in terms of Green O’ Brien Scoring system.^18^ Measurements were taken by one observer (JIK) at the conclusion of 12 weeks follow-up. This scoring system is based on pain, functional status, range of motion and grip strength. The patients were labeled as excellent; if a total score of 90 – 100 points, good; if a total score of 80 – 89 points, fair: if a total score of 65 – 79 points and poor; if a total score was< 65. Main outcome variable was excellent outcome and was presented as frequency distribution table. Performa designed especially for this study was used for data collection. The collected information was entered in SPSS version 18.00 and arranged through it. The two groups were compared with each other. Chi-square test was applied for test of significance. p-value< 0.05 was taken as significant.

## RESULTS

Mean age of patients in Group-A and Group-B was 36.13±9.81 and 44.73±7.86 years. Minimum age in Group-A and Group-B was 26 and 25 respectively. While maximum age in both treatment groups was 55 years. In Group-A Male: Female ratio was 2:1 & Group-B it was 1:0.87.

**Fig.1:**
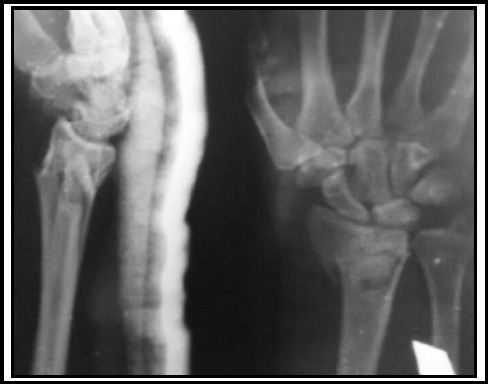
(Fixation with K-Wires) Male of 37 years sustained Fernandez type II fracture to the right wrist after a road traffic accident. AP. and Lat. views of injury film.

**Fig.2:**
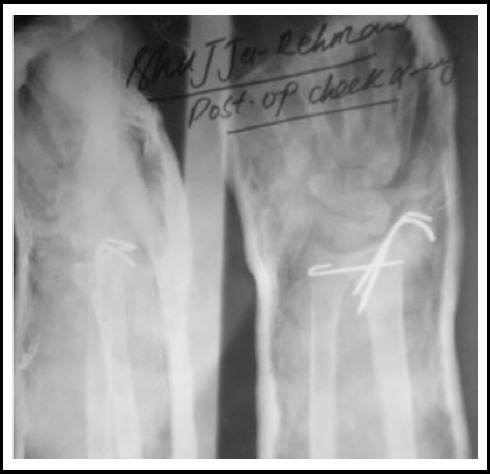
Post Operative Radiograph with AP & Lat View.

**Fig.3:**
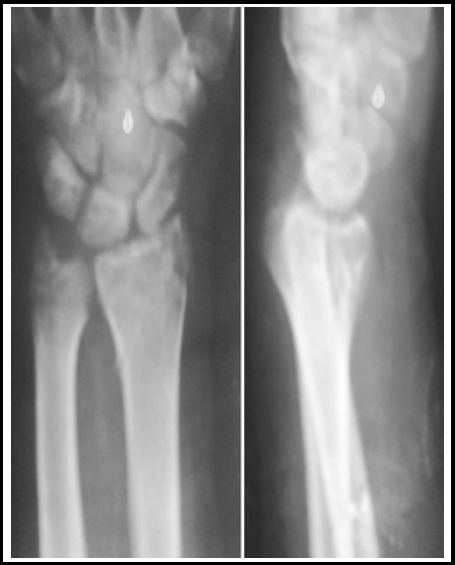
Radiographs after removal of K-Wires showing Fracture Union.

**Fig.4:**
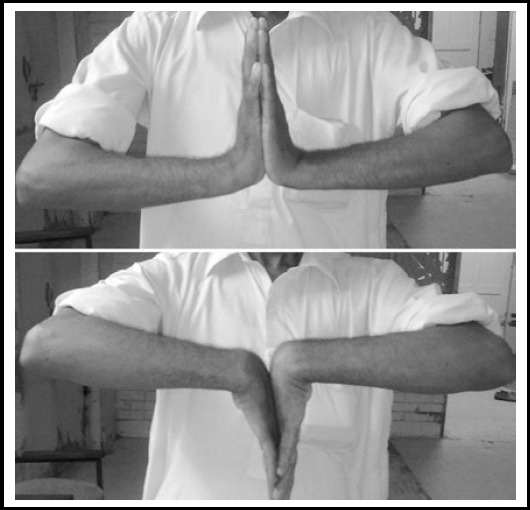
Demonstration of Range of Movement (Extension & Flexion of the Wrist).

**Fig.5:**
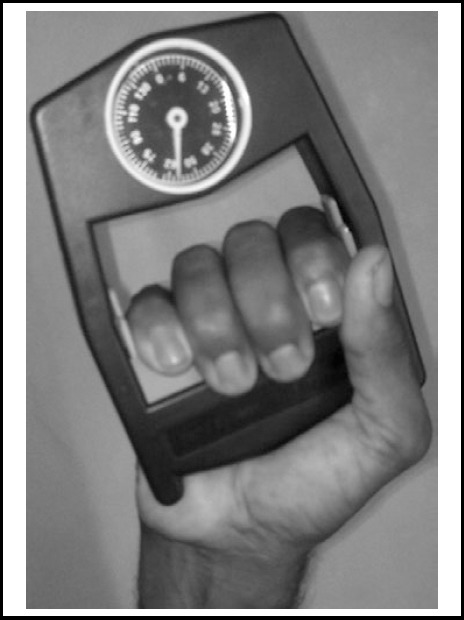
Measuring Grip Strength with Dynamometer.

Most of the patients fell in Fernandez classification II/III (Table-I). Grip strength was measured by grip strength dynamometer. In Group-A 12(86.67%) patients had excellent and 3(13.33%) patients had good outcome. While in Group-B 8(53.33%) patients had excellent and 7(46.67%) patients had good outcome (Table-II). The functional outcome criteria was based on Modified clinical scoring system of Green and O’Brien. According to p-value final outcome was significantly associated with treatment groups. Among Group-A patient’s final outcome was excellent in 86.67% patients while in Group-B only 53.33% patients had excellent outcome Fig. 1-5. Our results clearly favor use of Krischner’s Wires for intra articular distal radius fractures. No notable complications were noted in any of the cases except some minor wound problems.

**Table-I:** Fracture classification in treatment groups.

		*Group-A*	*Group-B*
Fernandiz Type	II	9	10
	III	6	5

Total	15	15	

Group-A=Krischner’s Wires Group-B= T Plate.

**Table-II:** Functional outcome of both treatment groups.

	*Group-A(p-value)*	*Group-B(p-value)*
Excellent	13(86.67%)	8(53.33%)
Good	2(13.33%)	7(46.67%)
Fair	0(0%)	0(%)
Poor	0(0%)	0(%)

Total	15	15

Group-A=Krischner’s Wires Group-B= T Plate Chi-Square Test=3.96 p-value=0.0463

## DISCUSSION

Rozenthal et al. 2009^2^ have shown by randomized control trials that open reduction and internal fixation with simple and locking implants were usually superior to both external fixation and percutaneous K-wiring when judged by radiological parameters, functional results and occurrence of complication. At inclusion all the patients (20-55 yrs) with intra-articular fractures of the distal radius (Fernandez type II and III) were included along with isolated distal radius fracture, including Graham TJ criteria for Post-op and either gender. we are talking about the study under reference All those with open fractures, fractures older than two week, with dislocation of wrist, arthritic wrists, previous fractures of wrist or elbow, neurovascular injury and Chauffeur’s fracture were excluded we are talking about the study under reference.

Beharrie et al. in 2004^19^ published a study comparing these two methods. They showed a clear advantage of Krischner’s Wires fixation over T-plate method. Avoided for want of brevity Radwanetal.^6^ Hull et al.^20^ have not found the wire fixation to be superior to the plate fixation technique. Our observations favor study K Wire to be more effective giving excellent results in terms of functional outcome as compared to that of T-plate. The main difference was Hull and Peter used volar locking plates while we used simple T-plate. But as in any operative procedure the skills of the surgeon is a major confounding variable between our two studies as each surgeon differs in his skills. Kiernan C in his study^21^ compared outcomes in those treated with volar locking plate to those undergoing manipulation and Kirschner’s wire fixation in the 20-65 year population. Their results are contrary to ours but the outcome measured at the end of their work was radiological restoration of articular surface unlike ours.

We feel that k-wire group produced better results regarding pain, functional outcome and disability in the end although these were not our measured outcomes. The internal-fixation group had more complications like swelling stiffness of fingers and stitch abscesses. Lesser time for operation and lower cost also favored Percutaneous k-wire stabilization. Our study has a very short follow-up duration and is limited to study of one outcome only. It would be prudent to base ones conclusions upon a meta analysis or a large scale study in this matter.

## CONCLUSION

Percutaneous Kirschner’s wires appear more effective as compared to T-Plate fixation in terms of functional outcome for treating intra-articular distal radius fractures.

### Author`s Contribution

The study was conducted in partial fulfillment of MS Orthopedics by **JIK** under supervision of **OA**.

**JIK** and **TM** collected data while **JIK** and **OA** analyzed it.

**JIK** and **FNH** prepared the manuscript.
